# The Impact of Klotho in Cancer: From Development and Progression to Therapeutic Potential

**DOI:** 10.3390/genes16020128

**Published:** 2025-01-23

**Authors:** Miguel A. Ortega, Diego Liviu Boaru, Diego De Leon-Oliva, Patricia De Castro-Martinez, Ana M. Minaya-Bravo, Carlos Casanova-Martín, Silvestra Barrena-Blázquez, Cielo Garcia-Montero, Oscar Fraile-Martinez, Laura Lopez-Gonzalez, Miguel A. Saez, Melchor Alvarez-Mon, Raul Diaz-Pedrero

**Affiliations:** 1 Department of Medicine and Medical Specialities, Faculty of Medicine and Health Sciences, Network Biomedical Research Center for Liver and Digestive Diseases (CIBEREHD), University of Alcalá, 28801 Alcala de Henares, Spain; diego.boaru@edu.uah.es (D.L.B.); diegodleonoliva01@gmail.com (D.D.L.-O.); patriciadecastro1999@gmail.com (P.D.C.-M.); anaminayabravo@gmail.com (A.M.M.-B.); silvebarrena@gmail.com (S.B.-B.); cielo.gmontero@gmail.com (C.G.-M.); oscarfra.7@gmail.com (O.F.-M.); msaega1@oc.mde.es (M.A.S.); mademons@gmail.com (M.A.-M.); 2 Ramón y Cajal Institute of Sanitary Research (IRYCIS), 28034 Madrid, Spain; laura.lgonzalez@uah.es (L.L.-G.); raul.diazp@uah.es (R.D.-P.); 3 Department of General and Digestive Surgery, Príncipe de Asturias, University Hospital, 28805 Alcala de Henares, Spain; 4 Department of Surgery, Medical and Social Sciences, Faculty of Medicine and Health Sciences, University of Alcalá, 28801 Alcala de Henares, Spain; 5 Pathological Anatomy Service, Central University Hospital of Defence—UAH Madrid, 28801 Alcala de Henares, Spain; 6 Immune System Diseases-Rheumatology, Oncology Service an Internal Medicine (CIBEREHD), University Hospital Príncipe de Asturias, 28806 Alcala de Henares, Spain

**Keywords:** α-Klotho, β-Klotho, γ-Klotho, cancer, breast cancer, colorectal cancer

## Abstract

Klotho, initially identified as an anti-aging gene, has been shown to play significant roles in cancer biology. Alongside α-Klotho, the β-Klotho and γ-Klotho isoforms have also been studied; these studies showed that Klotho functions as a potential tumor suppressor in many different cancers by inhibiting cancer cell proliferation, inducing apoptosis and modulating critical signaling pathways such as the Wnt/β-catenin and PI3K/Akt pathways. In cancers such as breast cancer, colorectal cancer, hepatocellular carcinoma, ovarian cancer, and renal cell carcinoma, reduced Klotho expression often correlates with a poor prognosis. In addition, Klotho’s role in enhancing chemotherapy sensitivity and its epigenetic regulation further underscores its potential as a target for cancer treatments. This review details Klotho’s multifaceted contributions to cancer suppression and its potential as a therapeutic target, enhancing the understanding of its significance in cancer treatment and prognoses.

## 1. Introduction

The *Klotho* gene was discovered in 1997 by Kuro-o et al. and was named after one of the Fates, Klotho, the Greek goddess who spins the thread of life. It was identified in a mouse strain as a gene that mutates and develops multiple aging-like phenotypes [1] such as hypogonadotropic hypogonadism, growth degradation, rapid thymic involution, sarcopenia, vascular calcification, skin atrophy, cognition impairment, pulmonary emphysema, osteopenia, motor neuron degeneration, and hearing disturbances [2,3,4,5]. Conversely, overexpression of this gene contributes to an increased lifespan [6]. Due to its significant role in aging processes, Klotho (KL) has been proposed as a potential biomarker of aging, with its function linked to various age-related diseases and conditions.

Aging involves a gradual decline in vital physiological functions that are necessary for survival and reproduction. This process involves dynamic changes in biological, physiological, environmental, psychological, behavioral, and social factors [7]. The differentiation between aging hallmarks is challenging due to their interconnected nature, rendering them not mutually exclusive. Consequently, emphasis is placed on the molecular, cellular, and systemic mechanisms underlying aging progression.

Aging is a risk factor for various types of cancer, whose incidence peaks at 85 years old [8]. After age 90, both cancer incidence and mortality decline, and beyond age 100, these rates drop to less than 5% of the overall morbidity and mortality, which is in contrast to increased cases of respiratory, infectious, and neurodegenerative diseases [8,9]. Aging is characterized by genomic instability, telomere attrition, epigenetic changes, chronic inflammation, cellular senescence, mitochondrial dysfunction, etc. [10,11,12]. Each hallmark of aging gradually emerges over time and can accelerate aging when exacerbated.

Klotho, a key regulator in kidney health, is strongly influenced by DNA methylation, a process controlled by enzymes like DNMT1, DNMT3a, and DNMT3b. Hyperactivity of these enzymes in conditions like chronic kidney disease (CKD) leads to Klotho promoter hypermethylation, suppressing its expression and worsening kidney dysfunction. Uremic toxins, inflammation, and oxidative stress exacerbate this epigenetic silencing [13].

Therapeutic strategies targeting DNA demethylation have shown promise in restoring Klotho levels. Agents like decitabine, Rhein, and hydrogen-rich saline reverse promoter hypermethylation, alleviating kidney damage and fibrosis. Additionally, hydroxymethylation by TET proteins, particularly TET3, plays a critical role in demethylating the Klotho promoter, with innovative methods like CRISPR/Cas9 enhancing this process [14].

Restoring Klotho expression improves kidney health by reducing fibrosis, oxidative stress, and inflammation while mitigating complications such as vascular calcification and hypertension. Thus, targeting epigenetic mechanisms offers a promising approach for treating kidney diseases [15].

Cancer and aging share characteristics such as the accumulation of DNA damage, which contributes to both processes. DNA damage and genomic instability are also common in most cancers, leading to increased numbers of mutations due to defective genome maintenance systems [16,17].

In this sense, KL also has a post-translational action. Post-translational protein acetylation, regulated by HATs (histone acetyltransferases) and HDACs (histone deacetylases), has an important role in epigenetic regulation by modulating chromatin structure and gene transcription. Acetylation promotes gene expression by loosening chromatin, while deacetylation compacts chromatin, silencing genes. Although extensively studied in cancer, the role of protein acetylation in kidney diseases is emerging as significant [18,19].

In AKI and CKD, aberrant HDAC activity contributes to KL repression, a key factor in kidney health. HDAC1 and HDAC2, elevated in AKI, interact with NF-κB to suppress Klotho transcription. HDAC inhibitors, like TSA (trichostatin A) and valproate, reverse this suppression, mitigating AKI and its progression to CKD. Similarly, TSA alleviates Klotho loss in CKD by enhancing PPARγ acetylation [20,21].

Among HDAC isoforms, HDAC3 is a significant driver of kidney damage. Upregulated by TGFβ, HDAC3 forms a repressor complex with NcoR and NF-κB, inhibiting Klotho transcription. Selective inhibition of HDAC3 with RGFP966 derepresses Klotho, reducing renal fibrosis and injury. Furthermore, activation of SIRT1, a class III HDAC, by agents like SRT1720 and resveratrol restores Klotho levels, alleviates arterial stiffness, and reduces vascular calcification [22,23,24].

Emerging evidence suggests that less-studied epigenetic modifications also influence Klotho expression in kidney diseases, though data remains limited. In CKD mice with indoxyl sulfate (IS)-induced vascular calcification, Klotho mRNA hypermethylation was linked to overexpression of Mettl14, a methyltransferase-like protein. Additionally, inhibiting DOT1L, a histone H3/lysine 79 methyltransferase, alleviated renal fibrosis by restoring Klotho expression [25,26].

Histone modifications also play a role. Increased H3K27me3 levels, observed in aged and Klotho mutant mouse kidneys, were associated with reduced activity of the demethylase JMJD3. Inhibiting PRC2, the complex responsible for H3K27me3 trimethylation, decreased these levels and enhanced Klotho expression in renal tubule cells. Moreover, the long non-coding RNA MALAT1 contributes to glomerular endothelial damage under high-glucose conditions by epigenetically suppressing Klotho via the methyltransferase G9a [27].

Genetic defects contributing to genomic instability encompass loss of function mutations in caretaker genes as well as gain of function mutations within key signaling pathways [28,29].

**Figure 1:**
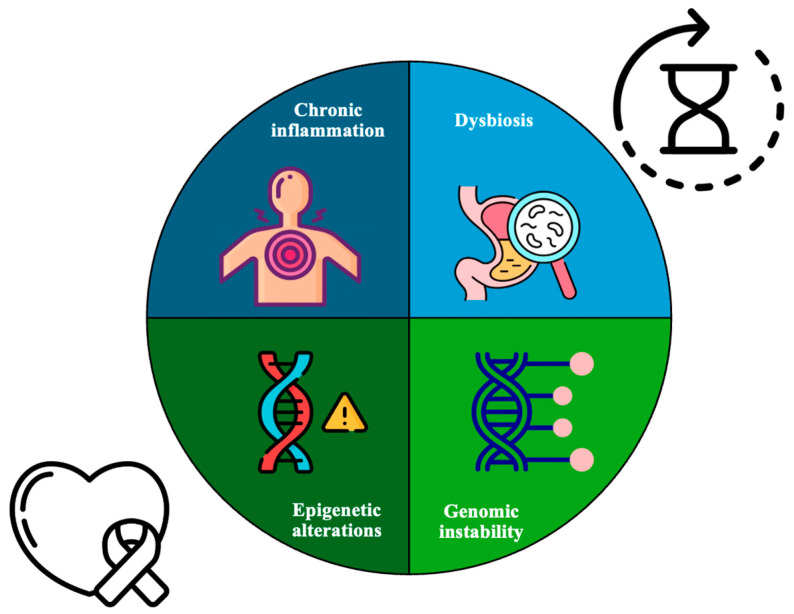
Shared critical hallmarks of aging and cancer including chronic inflammation, imbalances in the gut microbiota (dysbiosis), changes in gene expression (epigenetic alterations), and genomic instability. A key point is the accumulation of DNA damage, which plays an important role in both aging and cancer progression. This damage leads to increased rates of mutations because the body’s systems that normally maintain and protect the genome become less effective over time, contributing to the development and worsening of these conditions.

These mutations enable cancer cells to evade apoptosis, invade tissues, sustain angiogenesis, and maintain unlimited replication potential [30]. Aging and cancer share several key hallmarks that contribute to their progression and the resulting dysfunctions in patients (Figure 1).

Furthermore, it has become evident that Klotho could have a role in the progression of cancer. Thus, this review aims to summarize the evidence for the tumorigenic activity of all the members of the Klotho family through their association with certain mechanistic or distinctive aging factors that stimulate oncogenesis and the subsequent progression of tumors. At the same time, we also discuss other age-related mechanisms that can act to limit carcinogenesis.

## 2. Klotho Isoforms

Klotho is synthesized in the kidneys, particularly in the renal tubules. Research involving nephrectomized rodents and mice with kidney-specific genetic deletions of the gene Klotho has revealed that the kidney serves as the main source of circulating Klotho. It is produced by the proximal and distal convoluted renal tubules, with a notably higher production in the distal tubules [31,32]. Interestingly, Klotho appears to have distinct effects on the proximal and distal tubules [5]. In the proximal tubule, it exerts a phosphaturic effect and inhibits vitamin D production, whereas in the distal tubule, it has a role in facilitating the reabsorption of calcium [33].

It is also detected in other tissues such as the brain (choroid plexus, cerebrospinal fluid, and neurons), pancreatic β cells, blood vessels, and skin [34]. Recent research has even identified its expression in circulating cells in the peripheral blood [35], and it appears that Klotho is excreted into the urine.

**Figure 2:**
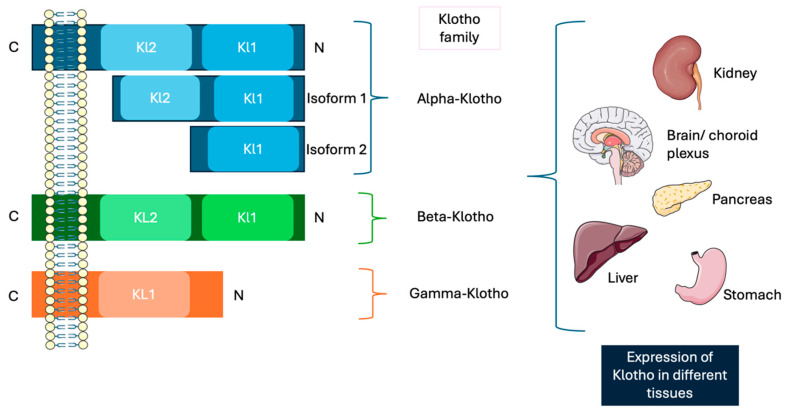
Three members of the Klotho family. α-Klotho, the first member studied, exists in two isoforms: Isoform 1 and Isoform 2. Then, β-klotho was discovered, and finally, γ-Klotho, which is the least studied member. Klotho is predominantly expressed in the kidney, although it is not exclusive to this organ. It is also present in other tissues such as the brain, liver, and stomach, suggesting a broader distribution across various bodily tissues.

Klotho is part of a family of single-pass transmembrane proteins and there are three isoforms of Klotho: α-, β-, and γ-Klotho [36,37] (Figure 2).

The discovery of this protein family was through α-*klotho*, which is located on human chromosome 13, in the q13.2 region in the long arm. It consists of five exons and its corresponding protein consists of an extensive extracellular domain, linking with a transmembrane domain and a small 11-residue intracellular C-terminal domain [38]. The extracellular domain contains two homologous domains known as KL1 and KL2, which are generated through splicing of the full-length transcript and are separated by the metalloproteases (such as ADAM-10 and ADAM-17). This cleavage leads to the release of a soluble form of Klotho [39], the main functional form in the circulation, which can be detected in blood, urine, and cerebrospinal fluid [40,41]. The soluble form of Klotho (s-Klotho) has been identified as a circulating factor that exerts protective effects against a range of systematic conditions, including chronic kidney disease, interstitial lung disease, and cardiovascular disorders [42,43,44,45]. Moreover, it has been shown to intricately regulate oxidative stress, endothelial function, cellular senescence, and apoptosis. Growing evidence underscores the protective role of s-Klotho in numerous pathological processes. For instance, one study enhanced its anti-apoptotic and anti-senescent properties in human umbilical vascular endothelial cells, as well as its ability to increase antioxidant defenses within the vascular system [46]. Additionally, s-Klotho mitigates endothelial dysfunction by modulating nitric oxide bioavailability, thereby offering protection against cardiovascular conditions such as atherosclerosis [47]. Furthermore, it has a pivotal role in calcium homeostasis by regulating epithelial calcium concentrations, thereby contributing to the preservation of normal renal function [48].

α-klotho has two human isoforms. The full-length protein is a 130 kDa single-pass transmembrane protein, composed of 1012 amino acids, which includes a signal sequence, KL1, KL2, and a short cytoplasmatic tail.

The second isoform, a 62 kDa protein composed of 549 amino acids, is produced via alternative splicing and exists as a secreted soluble protein known as soluble α-Klotho. This isoform only contains the SS domain and KL1, with the terminal 15 residues replaced by SQLTKPISSLTKPYH [49]. Although the secreted isoform predominates and circulates within plasma, its exact biological role remains unclear. In contrast, the full-length α-Klotho is closely associated with aging and the regulation of phosphate homeostasis. Acting as a coreceptor in conjunction with the FGF receptor, α-Klotho facilitates the activity of FGF23, a hormone produced in bone tissue [50]. FGF23 governs phosphate excretion and suppresses vitamin D synthesis. This interaction relies on α-Klotho to enable affinity binding of FGF23 to its receptor FGFR1, creating a binary complex that functions as the physiological receptor of FGF23 [51,52].

In addition, α-Klotho exhibits diverse pleiotropic roles within tissues, encompassing protection against oxidative damage, suppression of apoptosis and fibrogenesis, and facilitation of angiogenesis and vascularization, as well as vasculoprotective properties. Moreover, it regulates stem cell proliferation by modulating Wnt signaling pathways, all of which collectively may underline its potential anti-aging effects [53,54,55]. It influences these phenotypes by inhibiting several signaling pathways, such as the insulin-like growth factor receptor 1 (IGF-1R), fibroblast growth factor (FGF), transforming growth factor β (TGFβ), Wingless-related integration site (WNT), and PI3K/Akt pathways [56,57].

The circulation of Klotho directly influences tissues and cells lacking Klotho expression, providing insight into why mutations in the Klotho gene lead to widespread aging phenotypes. This circulating form of Klotho can arise either through alternative RNA splicing, which generates secreted Klotho, or via proteolytic cleavage of the transmembrane form, producing soluble Klotho. Consequently, Klotho could act as a hormone; however, its binding sites or receptors remain unidentified [58]. Future studies should focus on identifying and characterizing other Klotho receptors and investigating its downstream signaling pathway [59].

*β-Klotho* is located on chromosome 4 in humans, in the p16.3 region, and 41% of its amino acids sequence is homologous to that of α-Klotho [60]. It is a type I transmembrane protein and has a molecular weight of 130 kDa, made up of 1044 amino acids [61]. It consists of five exons and four introns, with a complex structure that consists of a signal sequence, an extracellular ligand-binding domain, a single transmembrane region, and a small cytoplasmatic segment [62]. βKL1 and βKL2 show homology to repeats in enzymes of the glycoside hydrolase 1 (GH1) family [59]. Despite this resemblance, the functionalities of two highly conserved glutamic acid residues in GH1 differ. Interestingly, both the βKL1 and βKL2 domains contain a single glutamic acid, lacking the active site residues required for GH1 enzymatic activity [63]. β-Klotho is selectively abundant in adipose tissue, the liver, and the brain, making it a key mediator of the pleiotropic effects of FGFs, such as FGF19 and FGF21, in several metabolic pathways. These effects affect several processes, such as glucose and lipid metabolism as well as bile acid biosynthesis [64].

On the other hand, γ-klotho forms complexes with FGFR1b, FGFR1c, FGFR2c, and FGFR4, functionally enhancing FGF19, and is present in ocular, adipose, and renal tissues. It is encoded by the γ-klotho gene [65]. The protein comprises a truncated type I transmembrane protein with a β-sole-glucosidases-like extracellular domain and a comparably brief cytoplasmatic segment [66]. In general, this molecule is less studied compared to the other members of the Klotho family. However, more studies are needed to have a better understanding of the action of this protein.

All the important aspects are summarized in Table 1.

## 3. Pleiotropic Functions of Klotho in Cancer

As mentioned above, KL exhibits pleiotropic activity in tissues, and several studies have demonstrated its role in tumorigenesis and cancer progression, as well as cancer prognosis. Klotho is described as a tumor suppressor in several solid tumors and hematological malignancies [67]. This action may be due to its role as an inhibitor in the tumor initiation or progression stage.

One of the pathways that involves Klotho is the inhibition of Transforming Growth Factor-β (TGF-β) [68]. TGF-β has a wide range of actions associated with aging, such as promoting stem cell declines, fibrosis, cellular senescence, and various diseases associated with aging [69]. KL exerts its effects by binding to the type II TGF-β receptor (TβRII), which appears to block TGF-β and its associated signaling molecules [70].

Klotho protein delivery has been demonstrated to inhibit TGF-β activity, thereby protecting against renal fibrosis [71]. Through its union with the TGF-β receptor, KL can counteract the actions of TGF-β, adding a layer of complexity to its regulatory functions. This interaction can also trigger the action of non-canonical pathways, including the JNK, p38, MAPK, PI3K/Akt, Rho-like GTPase, and NF-κB pathways [72,73,74].

Klotho also has a role in the inhibition of the Wnt signaling pathway, which is important in embryogenesis, stem cell biology, cell fate determination, cell polarity, and migration [75]. In Klotho-deficient mice, premature aging is characterized by several pronounced phenotypes, including skin atrophy. These mice exhibit sparser hair compared to controls, accompanied by histological evidence of reduced hair follicle density. Also, the skin shows a reduction in stem cell numbers, an accelerated aging of progenitor cells, and a market elevation in Wnt protein levels and signaling activity. These findings suggest that KL is necessary for maintaining both the number and functionality of stem cells [76].

Coimmunoprecipitation studies have demonstrated that s-Klotho interacts with different members of the Wnt group (such as Wnt1, Wnt3, Wnt4, and Wnt5a). By binding to these proteins, KL blocks Wnt transcription and curtails its function in the skin. The increase in Klotho counteracts the action of both endogenous and exogenous Wnt, which are known to accelerate cell senescence in vitro and in vivo. Thus, overexpression of Wnt proteins may represent a pathogenic factor implicated in senescence, as Klotho acts as a secreted modulator of Wnt signaling [54].

In this context of Wnt activity and its effects on tissue aging, elevated Wnt signaling has also been observed in the degenerative skeletal muscle of aged mice. This hyperactivation impairs muscle regeneration and repair, leading to increased tissue fibrosis, for example, in the kidneys, and it works alongside TGF-β to induce epithelial-mesenchymal transition (EMT). By blocking Wnt signaling, Klotho mitigates these adverse effects [77]. Alterations associated with the proliferation of satellite cells from a myogenic to a fibrogenic lineage are observed, driven by the canonical Wnt pathway in senescent myogenic progenitors. Remarkably, this process can be attenuated by inhibitors of Wnt like DKK1 and sFRP3, indicating that this pathway interacts with fibrosis and muscle stem cell aging. Acting as an antagonist of Wnt, KL can restore the function of myogenic stem cells, facilitate muscle repair, and reduce fibrosis [78].

In contrast, findings from a mouse model of X-linked hypophosphatemia of this condition, characterized by a deficiency in the Wnt coreceptor LRP6 (low-density lipoprotein receptor-related protein 6) and the subsequent reduction in Wnt signaling, showed no alteration in FGF23-induced phosphaturia or deficits in bone mineralization, suggesting a different Wnt pathway involved in phosphate homeostasis [79].

On the other hand, sKL is involved in the regulation of insulin and IGF-1 signaling. Research by different investigators has shown it prevents the ligand-induced autophosphorylation of insulin and IGF-1 receptors in a dose-dependent fashion. Additionally, it inhibits downstream situations such as the tyrosine phosphorylation of insulin receptor substrate proteins (IRS-1 and IRS-2) and the interaction between the p85 subunit of PI3K and IRS proteins [80], therefore inhibiting insulin and IGF-1 signaling through sKL-enhanced survival outcomes and mitigated age-related pathologies in Klotho-deficient mice. The role of sKL extends to cancer, a condition closely associated with aging. Insulin/IGF-1 signaling influences cell growth, programmed cell death, and tumorigenesis development. One study reported that certain cancers are stimulated by IGF-1 or IGF-2 through endocrine, autocrine, or paracrine mechanisms. Another study demonstrated that diabetes treatments that involve insulin or insulin secretagogues elevate the likelihood of developing solid tumors [81]. At the molecular level, the binding of insulin and IGF-1 to their corresponding receptors triggers downstream signaling cascades, including the PI3K/Akt and MAPK/ERK1/2 pathways, which are necessary for normal cellular growth and maintenance. Disruption of these pathways leads to uncontrolled cell proliferation and the progression of cancer [55].

sKL is linked with oxidative stress responses in mammals by modulating pathways involving superoxide dismutase (SOD) and catalase. The study of Yamamoto et al. showed that sKL reduced paraquat-induced lipid oxidation and apoptosis in HeLa and CHO cells, indicating its role in conferring resistance to oxidative stress [55]. Mechanistically, sKL increases the expression of superoxide dismutase 2 (SOD2) by activating FOXO, thereby suppressing insulin/IGF-1/PI3K/Akt. The decreased phosphorylation of Akt and FOXO observed in HeLa cells further supported the increased activation in FOXO. Since SOD2 is necessary for the elimination of ROS, sKL seems to enhance resistance to oxidative stress. Similarly, catalase, another enzyme involved in ROS detoxification, has been linked to lifespan extension in mice when overexpressed [55].

Additional studies have shown that Klotho regulates phosphorylation-mediated signaling. This study reported that an excessive expression of Klotho minimizes IGF-1R phosphorylation and its downstream targets, such as ERK1 and ERK2, in MCF-7 breast cancer cells. This inhibitory effect was also observed in insulin signaling pathways. Furthermore, Klotho knockdown in these cells led to elevated Akt phosphorylation-linked IGF-1 activation. While Klotho inhibited IGF-1 and insulin signaling in certain cells, it enhanced DGD signaling in breast cancer cells [82].

Some studies explored the involvement of Klotho in FGF signaling, showing that Klotho treatment phosphorylated the FGF receptor substrate two and activated both the Akt and ERK1/2 pathways in rat oligodendrocyte precursor cells. These pathways were found to be important in OPC maturation, as inhibition of Akt or ERK reduced Klotho effects, with Akt inhibition completely abolishing them. Also, Klotho treatment increased the phosphorylation and activity of STAT3, further promoting OPC maturation [83].

The activation of FGFRs is associated with the proliferation, survival, and angiogenesis of cancer cells [84]. When overexpressed, this pathway leads to increased cancer cell proliferation with the decline of apoptosis, allowing tumor cells to thrive [85]. In breast cancer, Klotho overexpression promotes the FGF pathway, further promoting cancer cell growth [86]. Conversely, in pancreatic cells, Klotho expression inhibits ERK1/2, which contributes to the downregulation of cancer cell proliferation [87]. Additionally, silencing β-Klotho in hepatocarcinoma cells suppresses the phosphorylation of ERK1/2 and Akt, which are signaling molecules for cell survival and proliferation [88]. In prostate cancer, Klotho may act as an endocrine growth factor, facilitating the progression of the disease. There is also evidence suggesting that Klotho can function as a co-receptor for FGF19; this mechanism involves using different pathways to influence the progression of cancer cells [89].

All these actions of Klotho are summarized in Table 2.

### 3.1. Acute Myeloid Leukemia

Acute myeloid leukemia (AML) is a malignant neoplasm originating from precursor stem cells of the myeloid lineage. Most AML cases arise from genetic alterations, including chromosomal abnormalities and gene mutations, with only a small subset linked to prior chemotherapy and chemical exposures [90]. For example, Shibayama et al. demonstrated that in AML patients, miR-126-5p inhibits the expression of α-Klotho by directly targeting it, leading to increased phosphorylation of Akt, which is known to play a role in the apoptosis of cancer. This suggests that Klotho expression is necessary for conferring resistance against this pathology. However, there is limited information available about Klotho’s role in this condition, with the focus mainly on the overexpression of the miRNA [91]. Further studies are necessary to better understand and enhance the therapeutic potential of Klotho in treating this pathology.

### 3.2. Bladder Cancer

Bladder cancer (BCa) is the most common malignancy of the urinary tract; 549,393 new cases were diagnosed worldwide in 2018 [92]. Hori et al. demonstrated that γ-Klotho plays a role in the growth of human UCB. The expression level of γ-Klotho is linked to cell proliferation, apoptosis, and EMT, and high levels can create a conducive environment for tumor survival and expansion. An enhanced understanding of the roles of γ-Klotho, along with those of α-Klotho and β-Klotho, could provide new therapies and diagnostic methods for UCB [93].

### 3.3. Breast Cancer

Breast cancer has various manifestations among women; 268,670 new cases were reported in the United States in 2018 [94,95,96]. The precise mechanism initiating this process is not understood [97]. In research to better understand and treat this malignancy, Klotho was found to play a role in the many physiological pathways affected by this cancer. Furthermore, γ-Klotho is overexpressed in more than 60% of TNBCs and has a poor prognosis. γ-Klotho is expressed in a subset of TNCB cell lines, where it promotes cell growth [98].

Trošt et al. demonstrated that γ-Klotho contributes to the resilience of these cells, as its depletion led to persistent EK activation, cell cycle arrest, and programmed cell death [98]. Additionally, increased oxidative stress was observed in γ-Klotho-depleted cells, indicating that γ-Klotho has a role in enabling cancer cells to cope with oxidative stress, thereby making its expression essential for their survival [98]. This research suggests that γ-Klotho could be used as a biomarker, but more investigation is necessary.

Rubinek et al. proved through immunohistochemistry (IHC) analysis that Klotho protein expression is higher in normal tissue samples in contrast to atypical ductal hyperplasia samples [99]. Furthermore, methylation of KL promoter was identified in five breast cancer cells and a portion of 8 out of 23 breast cancer samples [99]. This suggests that the reduction or absence of KL expression could represent an initial step in the onset of breast cancer.

Ligumsky et al. focused on the tumor-suppressive functions of the KL1 and KL2 domains of Klotho. Elevated expression of KL or the KL1 domain suppressed colony formation in the breast cancer cell lines MCF-7 and MBA-MB-231, while the KL2 domain showed no such effect. The in vivo administration of KL1 was well tolerated and significantly reduced tumor growth in nude mice [100]. Further experiments showed that KL1 interacted with the IGF-1 receptor and inhibited the IGF-1 signaling pathway. Structural modeling and mutation studies revealed that mutated KL proteins maintained their tumor suppressor activity despite a reduced ability to influence FGF23 signaling [100].

Wolf et al. demonstrated that including Klotho expression reduced the growth of MCF-7 and MBA-MB-231 breast cancer cells. Conversely, suppressing Klotho in MCF-7 cells, which naturally produce it, led to increased cell growth. Also, introducing Klotho into these cells or applying soluble Klotho inhibited IGF-1 and insulin signaling pathways. This process additionally enhanced the activity of CCAAT/enhancer-binding protein β, a transcription factor that suppresses breast cancer growth and is negatively impacted by the IGF-1/Akt pathway. These results suggest that Klotho acts as a tumor suppressor, inhibitor of the IGF-1 pathway, and activates the FGF pathway in breast cancer [101].

Research on γ-Klotho has revealed its essential role in cell survival, its depletion results in ERK activation, and its role in cell cycle arrest, cell programmed death, and heightened oxidative stress, enhancing its role in regulating oxidative stress in cancer cells. These findings propose γ-Klotho as a biomarker, although additional research is required to confirm its significance.

### 3.4. Colorectal Cancer

Colorectal cancer has become a significant worldwide health tissue, with its mortality rates on the rise. While numerous factors contribute to its prevalence, including demographic shifts and lifestyle habits, much of its heritability remains poorly understood [102].

Liu et al. reported that stromal cells (WI-38 and HUVEC), either pretreated with doxorubicin (DOX) or in a state of replicative senescence, promoted colorectal cancer cell growth and invasion in vivo and in vitro. The exogenous administration of Klotho mitigated these pro-tumorigenic effects [103]. The key findings included the upregulation of the SASP gene CCL2 in senescent stromal cells, with higher CL2 levels resulting in significantly higher rates of CRC cell proliferation and invasion. Klotho was found to inhibit the activation of NF-κB during DOX-induced senescence, preventing the transcription of CCL2. High CCL2 levels or low Klotho expression in patient tumors correlated with poor survival. These findings show the activation of senescent stroma cells in CRC progression and suggest that Klotho can inhibit this process [103].

Xie et al. showed that Klotho expression is linked with Friend leukemia virus-induced erythroleukemia 1 (FLI-1); they both affect colony formation, invasion, and apoptosis, and the low expression of either one of them correlated with poor survival of the patient [104]. So, in this sense, a reduction in the expression of the Klotho gene is linked to malignancy formation in different cancers, such as colon cancer.

Gunes et al. demonstrated that the interaction of Klotho with DR4 and DR5 induced apoptosis in cancer cells, reducing their proliferation. This study explored the downstream effects of overexpressing the *Klotho* gene, which has an antitumor effect on resistant human colon cancer cells, focusing on its action on TRAIL death and decoy (DcR1 and DcR2) receptors. The results showed that overexpressing the Klotho gene in Caco-2 cells sensitized the TRAIL death receptor DR4 and suppressed cell proliferation by inducing apoptosis [105].

Additionally, Sariboyaci et al. showed that KL could be a therapeutic agent for adjuvant therapy for human colorectal adenocarcinoma. It selectively induces apoptosis in cancer cells without affecting healthy colon cells [106].

Gene expression analysis by Rubstein et al. revealed that both Klotho and KL1 expression amplified the unfolded protein response (UPR), as evidenced by elevated levels of spliced XBP1, GRP78, and phosphorylated elF2α. Suppressing the UPR partially diminished the function of Klotho as a tumor suppressor. This study, for the first time, established a connection between the UPR pathway and the role of Klotho in cancer [107].

Conclusively, Li et al. observed a marked reduction in KL expression within human colon cancer, correlating with tumor invasiveness and Duke staging. Remarkably, enhanced Klotho expression curtailed both tumor growth and invasion by impeding the IGF1R-mediated PI3K/Akt pathway in colon cancer cells. This observation implies that KL could conceivably act as a therapeutic target for managing colon cancer [108].

Klotho mitigates tumor progression and enhances survival. It counteracts the pro-tumor effects of senescent stroma cells, reduces cell growth and invasion, and induces apoptosis in cancer cells by interacting with TRAIL death receptors. The overexpression of KL selectively targets cancer cells for apoptosis, while its reduction is linked with a poor prognosis.

### 3.5. Esophageal Cancer

Esophageal cancer has two main types of expression: adenocarcinoma and squamous cell carcinoma. Some elements can increase the chance of developing esophageal cancer, like age, tobacco and alcohol use, Barrett’s esophagus, and obesity [109]. Tang et al. revealed the presence of Klotho and β-catenin in patients with esophageal squamous cell carcinoma. An inverse correlation was found between Klotho and β-catenin expression levels [110]. The patients with Klotho-positive tumors had longer survival rates, and Klotho was identified as a significant factor for a good prognosis. This suggests that KL could be a biomarker for predicting progression and prognosis in ESCC patients [110].

### 3.6. Gastric Cancer

Gastric cancer is the fifth most common cancer and the third leading cause of cancer deaths worldwide [111].

Yang et al. explored the effect of the BR2-SOX17 union protein on *Klotho* gene expression in this cancer. BR2-SOX17 was assessed through proliferation, colony formation, apoptosis, and invasion essays. The findings revealed that SOX17 upregulated *Klotho* gene expression in this cancer by binding to the Klotho gene promoter [112].

Wang et al. reported that miR-199a-5p expression was elevated in gastric cancer tissues and inversely correlated with circ-ITCH levels. The use of miR-199-5p mimics counteracted the metastasis inhibition induced by circ-ITCH overexpression and reduced Klotho expression in cancer. These results suggest that circ-ITCH inhibits the metastasis of this cancer by acting as a sponge for miR-199a-5p, thereby promoting Klotho expression [113].

Wang et al. revealed that demethylation with 5-aza-2′-deoxycytidine (Aza) led to an increase in KL expression. The KL promoter was found to be hypermethylated in gastric cell lines and a subset of primary gastric carcinoma tissues but not in normal gastric tissues [114]. These findings demonstrate the role of epigenetic inactivation of KL in the development of gastric cancer [114].

In conclusion, KL acts as a novel tumor suppressor gene that is epigenetically silenced in gastric cancer, and KL promoter could serve as a valuable predictor of prognosis for gastric cancer patients.

### 3.7. Hepatocellular Carcinoma

Hepatocellular carcinoma (HCC) represents a significant medical challenge as the most common primary liver cancer [115]. HCC is the sixth most common neoplasm and the third leading cause of cancer deaths [116]. Klotho acts as a tumor suppressor in hepatocellular carcinoma through several mechanisms.

Sun et al. demonstrated that KL acts as a tumor suppressor gene, and its increased expression hindered the growth of liver cancer cells by negatively regulating the Wnt/β-catenin signaling pathway. Therefore, KL might be a therapeutic target to treat liver cancer patients [117].

Xie et al. measured Klotho mRNA and protein levels in 64 HCC tumor tissues using real-time PCR and immunohistochemistry, respectively, and examined KL promoter DNA methylation using bisulfite-based PCR [118]. The study concluded that Klotho could act as a tumor suppressor in HCC, with hypermethylation and acetylation contributing to its loss of expression. The KL gene expression and the promoter DNA methylation predict a poor prognosis in HCC, suggesting that the *Klotho* gene might be a therapeutic target for hepatocellular carcinoma treatment [118].

Poh et al. proved that elevated KLB levels in hepatocellular carcinoma tissues support the elevated FGFR4 signaling in HCC that promotes development, which could serve as a novel biomarker for identifying patients who may benefit from anti-FGFR4 therapy [88]. The restricted tissue expression profile of KLB and the antiproliferative effects observed with KLB silencing make it a specific and therapeutic target for HCC therapies. The enriched liver stem cell-like population in response to extended KLB-FGFR4 expression highlights the need for further investigation to address potential drug resistance development [88].

### 3.8. Lung Cancer

Lung cancer originates in the lung parenchyma or bronchi. It is a leading cause of cancer-related deaths in the United States. Since 1987, lung cancer has caused more deaths in women than breast cancer [119].

Wang et al. showed that Klotho can reduce the cisplatin resistance of lung cancer tissues. The overexpression of Klotho increased apoptosis in resistant cells, while KL knockdown enhanced chemotherapy resistance [120]. The inhibition of the PI3K/Akt pathway with the inhibitor LY294002 minimized the cancer promotion seen with Klotho knockdown. These findings suggest that Klotho could improve chemotherapy effectiveness in lung cancer and target gene therapy in cases of cisplatin resistance [120].

Ibi et al. revealed that Klotho interacts in regulating EMT in lung squamous cell carcinoma. The transfection of Klotho into SQ5 lung cancer cells revealed that Klotho inhibited the mesenchymal marker N-cadherin, although it had no impact on other EMT markers like Snail, vimentin, or the epithelial marker E-cadherin [121].

Brominska et al. suggested that Klotho might be a positive predictor of survival in lung large cell neuroendocrine carcinoma (LCNEC), while nodal involvement has a negative value as a predictor [122].

### 3.9. Ovarian Cancer

Ovarian cancer is the fifth leading cause of cancer-related deaths among women in the United States, with approximately 140,000 deaths globally each year. This disease often presents with subtle symptoms, leading to limited treatment options at the time of diagnosis [123].

Lojkin et al. demonstrated that Klotho mRNA levels were reduced in most epithelial ovarian cancer (EOC) cell lines; its expression was high in normal ovaries but reduced in many high-grade papillary-serous adenocarcinomas of the ovaries, fallopian tubes, and peritoneum [124]. Reduced Klotho expression was associated with a wild-type BRCA status. Klotho decreased EOC cell viability, increased the sensitivity to cisplatin, and reduced mesenchymal marker expression. It also blocked the activation of the IGF-1 pathway and the transcriptional activity of the estrogen receptor. Reintroducing Klotho expression slowed the growth of EOC cells and suppressed key signaling pathways [124].

Yan et al. proposed that enhancing Klotho expression could potentially hinder tumor growth in animal models. In summary, Klotho serves as a powerful tumor suppressor in human ovarian cancer cells. A marked reduction in KL expression was observed in patients’ samples, while the overexpression of KL notably suppressed the proliferation of ovarian cancer cells in vitro [125].

### 3.10. Pancreatic Cancer

Pancreatic cancer is a leading cause of cancer-related deaths worldwide, with its global burden having more than doubled over the past 25 years [126]. Inherited genetic factors, which are not directly modifiable, have a substantial impact on pancreatic cancer risk [127]. Understanding the genetic changes will contribute to a better understanding of this pathology.

Rubinstein et al., employing an innovative genetic model with pancreatic Klotho knockdown and a Kras mutation, demonstrated that the lack of KL accelerates the onset of pancreatic ductal adenocarcinoma and shortens survival in mice. In a xenograft model, viral particles carrying the spliced KL isoform sKL inhibited pancreatic tumors [128]. These findings indicate that KL functions as a tumor inhibitor in PDAC and suggest that Klotho expression and DNA methylation could be prognostic markers [128].

Jiang et al. illustrated that in patients with PDAC, the downregulation of KL expression triggers hypermethylation, elevated levels of miR-504, and an increase in phosphor-IGF-1R, all of which showed an association with poorer survival outcomes and more advanced clinical and pathological stages [129]. Notably, treatment with a demethylation agent and miR-504 inhibitor led to a notable upregulation of KL expression, simultaneously reducing the invasive and migratory capabilities of BxPC-3 and Panc-1 cells. Hence, Klotho functions as a tumor suppressor, with its expression being intricately connected to both the progression and prognosis of PDAC [129].

### 3.11. Prostate Cancer

Prostate cancer affects men across all racial and ethnic groups and disproportionately leads to higher mortality rates among those from lower socioeconomic backgrounds, often due to late detection [130].

Feng et al. used low concentrations of exogenous FGF19 to improve the growth, invasion, adhesion, and colony formation of prostate cancer cells. Silencing FGF19 in prostate cancer cells expressing autocrine FGF19 reduced their invasion and proliferation in vitro and tumor growth in vivo [89]. Prostate cancer cells express α-Klotho and/or β-Klotho, both in vitro and in vivo, suggesting that other endocrine FGFs may also exert biological effects in prostate cancer. These findings suggest that therapies targeting FGFR signaling may be effective in treating prostate cancer [89].

In a separate study, Onishi et al. developed a xenograft model using a human CRPC cell line (PC-3) in male athymic mice to evaluate the therapeutic use of γ-Klotho in human prostate cancer. After a period of three weeks of using the treatment, the weight and the Ki-67 labeling index were lower in the γ-Klotho siRNA group and the combination therapy group compared to the control [131]. These results demonstrated that γ-Klotho expression is linked to resistance mechanisms in the study groups and posit γ-Klotho as a valuable diagnostic and therapeutic target for patients with CRPC [131].

### 3.12. Renal Cell Carcinoma

Renal cell carcinoma (RCC) is the most common form of urogenital cancer, with a mortality rate ranging from 30% to 40%, and it is more frequently diagnosed in men than in women [132].

Zhu et al. showed that Klotho can inhibit the PI3K/Akt/GDK3β/Snail pathway. Advanced RCC tissues showed lower Klotho expression and higher pAkt and Snail expression compared to localized RCC tissues. These findings suggest that KL acts as a tumor suppressor by inhibiting the PI3K/Akt/GDK3β/Snail pathway, which suppresses epithelial-mesenchymal transition, tumor migration, and invasion, which contributes to RCC progression [133].

### 3.13. Thyroid Cancer

Thyroid cancer ranks as the fifth most common cancer in women in the USA, with over 62,000 new cases in men and women reported in 2015 [134].

Dai et al.’s study aimed to investigate the effects and mechanism of KL in the human thyroid cancer cell lines FTC133 and FTC238. The overexpression of Klotho reduced cell proliferation and increased apoptosis, while Klotho silencing enhanced cell growth. High KL levels were associated with a low stanniocalcin 1 (STC1) level in both cell lines. Moreover, hSTC1 treatment counteracted the Klotho-induced inhibition of cell proliferation and promotion of apoptosis [135].

**Table 2:** Overview of the action of Klotho in various cancers.

Type of Cancer	In Vivo	In Vitro	Isoform	Pathways/Mechanism Involved	Reference(s)
Acute myeloid leukemia		x	α	In AML, miR-126-5p suppressed the expression of α-Klotho, resulting in elevated phosphorylation of Akt	[91]
Bladder cancer	x	x	γ	γ-Klotho was linked to cell proliferation, apoptosis, EMT, and growth of human UCB	[93]
Breast cancer		x	α, β, γ	γ-Klotho helped cancer cells manage oxidative stress Overexpression of Klotho or the KL1 domain inhibited tumor formation in the breast cancer cell lines MCF-7 and MBA-MB-231	[98,99,100,101]
Colorectal cancer	x	x	α	It has been demonstrated that Klotho inhibits the activation of NF-κB. Remarkably, overexpressing Klotho in Caco-2 cells sensitized the TRAIL death receptor DR4 and impeded cell proliferation by promoting apoptosis. Furthermore, an increase in KL expression suppressed tumor growth and invasion, primarily through the inhibition of the IGF1R-mediated PI3K/Akt pathway in colon cancer cells	[103,104,105,106,107,108]
Esophageal cancer		x	α	An inverse correlation was found between Klotho and β-catenin expression levels. Klotho was identified as a significant factor for a good prognosis	[110]
Gastric cancer		x	α	SOX17 promoted the expression of the Klotho gene in gastric cancer cells circ-ITCH suppressed gastric cancer metastasis by acting as a sponge for miR-199a-5p, thereby increasing Klotho expression	[112,113,114]
Hepatocellular cancer	x	x	α, β	The overexpression of Klotho curtailed the proliferation of liver cancer cells. Also, both KL gene expression and the methylation of its promoter DNA emerged as strong indicators of poor prognosis with HCC	[88,117,118]
Lung cancer	x	x	α	The inhibition of the PI3K/Akt pathway using the inhibitor LY294002 diminished the enhanced cancer growth observed with Klotho knockdown. Furthermore, transfecting Klotho into SQ5 lung cancer cells demonstrated its ability to suppress the mesenchymal marker N-cadherin	[120,121,122]
Ovarian cancer	x	x	α	Restoring Klotho expression slowed EOC cell growth and inhibited key signaling pathways. Klotho functions as a tumor inhibitor in human ovarian cancer cells	[124,125]
Pancreatic cancer	x		α, β	Klotho functions as a tumor suppressor in PDAC. Treatment with miR-504 inhibitor and a demethylation agent upregulated Klotho gene expression, while concurrently inhibiting the invasion and migration of BxPC-3 and Panc-1 cells.	[128,129]
Prostate cancer	x		α, β, γ	Prostate cancer cells exhibit the expression of both α-Klotho and β-Klotho, in vitro and in vivo, indicating that other endocrine FGFs might also influence the biological processes in this cancer.	[89,131]
Renal cell carcinoma	-	-	α	Klotho acted as a tumor suppressor by inhibiting the PI3K/Akt/GDK3β/Snail pathway	[133]
Thyroid cancer		x	α	High Klotho levels were associated with low stanniocalcin 1 (STC1) levels in FTC133 and FTC238 cell lines	[135]

## 4. Conclusions of Exploring the Tumor-Suppressive Role of Klotho in Cancer Progression

*Klotho* is a tumor suppressor gene with significant roles in inhibiting the progression of various cancers. The research suggests that Klotho functions by modulating several cellular signaling pathways, contributing to reduced cell growth and increased programmed cell death of cancer cells [67].

Klotho inhibits pro-tumoral vias, including the PI3K/Akt/GSK3β/Snail pathway, which is important for EMT, an important process in tumor invasion and migration in renal cell carcinoma and other cancers. By modulating signaling pathways, like the FGFR and TGF-β pathways, and the insulin/IGF-1/PI3K/Akt/mTOR axis, Klotho suppresses cell proliferation, induces apoptosis, reduces fibrosis, and activates antioxidant mechanisms, which underscores its therapeutic potential. Future research should focus on the precise mechanisms of Klotho’s actions in various cancer types.

Klotho levels are minimized or silenced in some of the cancers studied in this review. In the rest of the tumors, the patients with tumors that are positive for Klotho have a better prognosis. The decrease in Klotho occurs through mechanisms like those for other tumor suppressor genes, such as the ones mentioned above. For example, in hepatocellular carcinoma, KL suppresses Wnt/β-catenin signaling, reducing cell proliferation. The expression of Klotho can be affected by hypermethylation of the KL gene promoter, as observed in pancreatic and ovarian cancers, which is associated with a poor prognosis. Demethylation and inhibition of specific microRNAs can restore Klotho expression, suggesting that an epigenetic therapeutic approach could be used for some cancers. Furthermore, in lung and ovarian cancers, KL overexpression increases sensitivity to cisplatin chemotherapy, enhancing treatment efficacy and reducing drug resistance. Studies in animal models and cell cultures have demonstrated that KL overexpression significantly reduces tumor proliferation and promotes apoptosis. This was observed in lung, thyroid, prostate, and pancreatic cancers. Additionally, high levels of KL correlate with a better prognosis in various cancers, making it a biomarker for cancer progression and prognosis. On the other hand, exogenous administration of Klotho or its soluble form (sKL) has shown therapeutic effects in preclinical cancer models, suggesting it should be utilized as a treatment approach. In breast cancer, there are significantly lower Klotho levels compared to normal breast tissue. In this cancer, Klotho modulates the IGF-1 and FGF pathways, reducing tumor cell proliferation and enhancing apoptosis. In TNBC (Triple-Negative Breast Cancer), Klotho is overexpressed in a subset of TNBC cells and is necessary for cell survival. Targeting Klotho in these cells leads to increased oxidative stress, cell cycle arrest, and apoptosis, indicating its potential as a therapeutic target for TNBC. Finally, in colorectal cancer, Klotho suppresses the tumorigenic effects of senescent stromal cells by suppressing SASP factors and CCL2.

Frailty arises from the intricate disruption of interconnected biological systems, including chronic inflammation, energy imbalances, oxidative stress, endocrine dysregulation, sarcopenia, and malnutrition [136]. This complexity challenges clinical detection and intervention, underscoring the urgent need for accurate biomarkers to aid early diagnosis, prognosis, and treatment [137]. Recent advances in omics technologies have opened a promising avenue for identifying these biomarkers, offering valuable insights into the molecular basis of frailty [138]. Among them, the protein Klotho has emerged as a potential biomarker. Data from the InCHIANTI study, which followed more than 1400 participants for six years, revealed an inverse relationship between plasma levels of Klotho and the incidence of frailty, highlighting its potential for early intervention. Similarly, innovations in spatial cellular analysis are driving progress in oncology [139]. Techniques such as CosMx and MERSCOPE enable researchers to analyze gene expression at single-cell resolution, uncovering localized cellular responses to therapies [140,141]. These advances have revealed how tumor cells adapt to their microenvironments to foster drug resistance. For example, the Sparx model integrates pharmacogenomic data with spatial transcriptomics, offering insights into tumor heterogeneity and resistance mechanisms [142]. These findings hold promise for developing targeted therapies and optimizing treatment combinations.

Beyond frailty and oncology, multi-omic data integration is proving transformative in fields such as chronic liver disease (CLD) [143]. A global health challenge, CLD encompasses conditions such as hepatitis B, fatty liver disease, and hepatocellular carcinoma [144]. With artificial intelligence driving the digital transformation of omics analyses, researchers can now integrate bulk tissue and single-cell data with microbiome profiles, uncovering new molecular targets for precision therapies [143].

Microbial metabolomics further underscores the multi-omics integration. By combining genomics, transcriptomics, proteomics, and metabolomics, researchers are beginning to resolve the “metabolic dark matter” of microorganisms [145]. This approach not only improves our understanding of microbial communities but also paves the way for new therapeutic interventions [146]. Taken together, these advances illustrate the transformation of multi-omics platforms to unravel complex biological systems, address health challenges, and drive innovation. By bridging disciplines and adopting integrative approaches, getting closer to personalized solutions that address some of the most pressing problems in modern medicine.

In conclusion, Klotho has emerged as a potent tumor suppressor that acts by modulating vias related to cancer cell growth and survival, regulating epigenetic mechanisms, and increasing sensitivity to chemotherapy. Also, its function remains underexplored; more research is necessary to define the action of Klotho in cancer and its functions as a biomarker in cancer.

## Figures and Tables

**Table 1:** Overview of the Klotho isoforms.

	α-Klotho	β-Klotho	γ-Klotho
Human chromosome location	Chromosome 13	Chromosome 4	-
Full-length protein size	130 KDa/1020 aa	130 KDa/1044 aa	-
Receptor(s)	FGF23	FGF19 and FGF21	FGFR1b, FGFR1c, FGFR2c, FGFR4, and FGF19
Expression pattern	Kidneys and brain	Adipocytes, liver, and brain	Ocular, adipose, and renal tissues
Functions	Protection against oxidative stress, inhibition of apoptosis and fibrogenesis, promotion of angiogenesis and vascularization, and vasculoprotective functions	Regulation of several metabolic pathways, energy balance, and glucose and lipid homeostasis	Metabolic regulation and cellular protection

## Data Availability

No new data were created or analyzed in this study.
